# 

**Published:** 1869-09

**Authors:** 


### Books and Pamphlets Received.

The Science and Art of Surgery. By John Eric Erichsen, Senior Surgeon to University College Hospital, and Holme Professor of Clinical Surgery in the University College of London. From the Fifth English edition: Edited by John Ashurst, Jr., A. M., M. D., &c. Philadelphia: H. C. Lea, 1869. Received through Theo. Butler & Son.

A Treatise on the Diseases and Surgery of the Mouth, Jaws and Associate Parts. By James E. Garretson, M.D., D. D. S., late Lecturer on Anatomy and Surgery in the Philadelphia School of Anatomy, late Professor of the Principles and Practice of General Surgery in the Philadelphian Dental College, etc., etc. Illustrated with Steel Plates and numerous wood cuts. Philadelphia: J. B. Lippincott & Co., 1869. For sale by Breed & Lent.

Transactions of the Medical Society of the State of Pennsylvania at its Twentieth Annual Session, held at Erie, June, 1869. Published by the Society.

Carbolic Acid: Its Action and Uses. By Chas. F. J. Lehlbach, M. D., of Newark, N. J.

Surgery of the Cervix, in connection with the treatment of certain Uterine Diseases. By Thomas Addis Emmet, M. D., Surgeon-in-Chief of the New York State Woman's Hospital, etc., etc. From the Author.

Physical Culture in Amherst College. By Nathan Allen, M.D., Lowell, Mass. From the Author.

Report and Remarks on a third series of one hundred cases of Cataract Extraction by the Peripheric-Linear Method. By H. Knapp, M. D., late Professor of Ophthalmology and Surgeon to the Ophthalmic Hospital at Heidelberg, &c., &c. New York: Wm. Wood & Co.

Myxoma, or Hyperplasia of the Villi of the Chorion. By Alexander D. Sinclair, M. D., Physician to the Boston City Hospital, etc. From the Author.

Hygiene in its relations to Therapeusis. By Alfred L. Carroll, M. D., one of the Editors of the Medical Gazette, etc., New York.

Proceedings of the Texas State Medical Association Meeting, held at Houston, June 15th, 1869.

Council of the College of Physicians and Surgeons of Ontario. Rules, Regulations, etc , for the guidance of students in medicine. Toronto, August, 1869.

Catalogue of the Graduates of Jefferson Medical College from its organization. Philadelphia.

Annual Announcement of the Medical Department of the University of Louisville, Kentucky.

Annual Announcement of the Medical Department of Willamette University, Salem, Oregon.

Publishers and Stationers Trade List Directory for 1869. Advance Edition. Howard Challen, Philadelphia.

Literarischer Monatsbericht. A catalogue of new German Scientific and Literary publications. E. Steiger, 17 North William St., New York.

Price List of Microscopical Instruments. T. H. McAllister, 49 Nassau St., New York.

Catalogue of the Medical Publications of Lindsay & Blackiston, Phila.

The Illustrated Educational Bulletin. A. S. Barnes & Co., New York.

